# Potential Therapeutic Approaches for Cerebral Amyloid Angiopathy and Alzheimer’s Disease

**DOI:** 10.3390/ijms21061992

**Published:** 2020-03-14

**Authors:** Masashi Tanaka, Satoshi Saito, Takayuki Inoue, Noriko Satoh-Asahara, Masafumi Ihara

**Affiliations:** 1 Department of Physical Therapy, Health Science University, 7187 Kodachi, Fujikawaguchiko-machi, Minamitsuru-gun, Yamanashi 401-0380, Japan; 2 Department of Endocrinology, Metabolism, and Hypertension Research, Clinical Research Institute, National Hospital Organization Kyoto Medical Center, 1-1 Fukakusa Mukaihata-cho, Fushimi-ku, Kyoto 612-8555, Japan; taka2015.www@gmail.com (T.I.); nsatoh@kuhp.kyoto-u.ac.jp (N.S.-A.); 3 Department of Neurology, National Cerebral and Cardiovascular Center, 6-1 Kishibe-Shimmachi, Suita, Osaka 564-8565, Japan; saitou.satoshi.43m@kyoto-u.jp (S.S.); ihara@ncvc.go.jp (M.I.)

**Keywords:** Alzheimer’s disease, amyloid-β, antioxidants, cerebral amyloid angiopathy, cilostazol, glycation, inflammation, intramural peri-arterial drainage, taxifolin, triggering receptor expressed on myeloid cells 2

## Abstract

Cerebral amyloid angiopathy (CAA) is a cerebrovascular disease directly implicated in Alzheimer’s disease (AD) pathogenesis through amyloid-β (Aβ) deposition, which may cause the development and progression of dementia. Despite extensive studies to explore drugs targeting Aβ, clinical benefits have not been reported in large clinical trials in AD patients or presymptomatic individuals at a risk for AD. However, recent studies on CAA and AD have provided novel insights regarding CAA- and AD-related pathogenesis. This work has revealed potential therapeutic targets, including Aβ drainage pathways, Aβ aggregation, oxidative stress, and neuroinflammation. The functional significance and therapeutic potential of bioactive molecules such as cilostazol and taxifolin have also become increasingly evident. Furthermore, recent epidemiological studies have demonstrated that serum levels of a soluble form of triggering receptor expressed on myeloid cells 2 (TREM2) may have clinical significance as a potential novel predictive biomarker for dementia incidence. This review summarizes recent advances in CAA and AD research with a focus on discussing future research directions regarding novel therapeutic approaches and predictive biomarkers for CAA and AD.

## 1. Introduction

Cerebral amyloid angiopathy (CAA) is a cerebral small vessel disease that results from amyloid-β (Aβ) deposition in the cerebrovasculature [1,2,3,4,5]. CAA is associated with cerebrovascular alterations and an increased risk of intracerebral hemorrhage, which can cause cognitive impairment [1,2,3,4,5,6]. The frequency and severity of CAA increase with aging, and CAA is often accompanied by Alzheimer’s disease (AD) and vascular cognitive impairment [1,2,3,4,5,6]. However, predictive markers and effective treatments for CAA have not been established.

The two predominant forms of Aβ consist of 40 (Aβ_40_) or 42 (Aβ_42_) amino acids [7,8]. Aβ_40_ is characterized by vasculotropic accumulation, whereas Aβ_42_ preferentially deposits in parenchymal senile plaques and capillaries [4,5,9,10,11,12]. Although both types of Aβ are cytotoxic, a recent study demonstrated multiple mechanisms underlying Aβ_42_-induced neurotoxicity. Aβ_42_ aggregates generate reactive oxygen species (ROS) and disrupt the neuronal membrane, thereby impairing neuronal metabolic integrity and synaptic function [7,8]. Although not much is known about the molecular mechanism of action of Aβ_40_ in comparison with Aβ_42_, Aβ_40_ deposited within cerebral vessels is strongly implicated in cerebrovascular dysfunction [9,10,11,12]. These compelling findings suggest Aβ as a therapeutic target for CAA and AD.

One serious issue of concern is that new drugs have not been approved for AD treatment in the past 15 years, despite extensive studies to develop therapeutics targeting Aβ accumulation [13]. Some candidate drugs that inhibit Aβ production, such as inhibitors of the proteolytic enzymes β- and γ-secretases, did not improve cognitive outcomes in spite of Aβ reduction in the brain. Rather, they exacerbated cognitive function deficits, possibly because of off-target effects [13]. Other drugs based on anti-Aβ immunotherapy reduced brain Aβ plaques, but did not result in cognitive benefits [13,14]. Particularly in AN-1792-vaccinated AD patients, cerebrovascular Aβ accumulation and CAA were concomitantly exacerbated with reductions in parenchymal Aβ plaques [12,15,16]. These findings may suggest that Aβ accumulation is a by-product, rather than a cause, of the AD process. This concern indicates the significance of identifying alternative strategies for targeting Aβ [13].

Recent basic and clinical studies have provided further evidence to support targeting Aβ as a therapeutic strategy for CAA and AD. These studies have elucidated the issues that need to be addressed for effective CAA and AD treatment. We recently provided the first evidence that in a mouse model of CAA, oral administration of taxifolin, a natural bioactive flavonoid, prevented cognitive impairment through pleiotropic beneficial effects [3,4]. Furthermore, a potential novel predictive blood marker for the development of dementia was recently identified based on a population-based longitudinal study [17]. This biomarker is a soluble form of triggering receptor expressed on myeloid cells 2 (TREM2), a protein implicated in the pathogenesis of neurodegenerative diseases [18,19]. Here, we review recent advances in CAA/AD research and discuss future research directions for developing effective treatments and predictive biomarkers for CAA and AD.

## 2. Pathophysiological Significance of the Aβ Drainage System

### 2.1. Intramural Peri-Arterial Drainage Pathway

Previous studies suggest that the antibody-solubilized, senile plaque-derived Aβ is redeposited in the cerebral vasculature and aggregates in CAA [20,21,22]. Therefore, eliminating Aβ from the brain and suppressing its production should be more significant than previously thought [12,23,24]. Several pathways have been proposed for Aβ drainage from the brain. A recent study demonstrated that the Intramural Peri-Arterial Drainage (IPAD) pathway is one of the major exit routes for Aβ from the brain [25]. The IPAD pathway is formed by the basement membranes of vascular smooth muscle cells (VSMCs) in the artery walls [25,26,27]. It is a physiological lymphatic drainage pathway for interstitial fluid and solutes from the brain [25]. To investigate Aβ elimination pathways from the brain, the authors conducted meticulous tracer experiments in which they injected soluble fluorescent Aβ into the cerebrospinal fluid (CSF) of the cisterna magna of mice. The temporal delivery profiles of Aβ were examined using multi-labeling immunocytochemistry and confocal microscopy analysis [25]. They found that the Aβ tracer was distributed in the spinally arranged smooth muscle cell basement membranes in the tunica media of arteries within the cerebral cortex [25]. This location precisely corresponded to the IPAD pathway [25,28,29,30]. Furthermore, the pattern of Aβ distribution was the same as that of Aβ deposition observed in CAA [25,28,29,30]. These results suggest that the IPAD pathway is a major route for Aβ drainage from the brain. Therefore, dysfunction of this pathway could be closely implicated in the pathogenesis of neurodegenerative diseases [25]. It is also suggested that increasing age-related risks of CAA are related to the IPAD pathway impairments. This would further promote a vicious cycle between Aβ accumulation in the brain and IPAD pathway dysfunction, especially in elderly individuals. In this respect, these findings raise new issues for further investigation, such as the mechanism of action of Aβ elimination along the IPAD pathway.

A recent study based on in silico analyses provided the first theoretical evidence of the mechanisms underlying the IPAD pathway [31]. To identify the motive force of the IPAD pathway, the authors developed a novel multiscale model of arteries that coupled two models. The arterial wall model was used for the elastic response of a middle cerebral artery, and the basement membrane model was used for the fluid flow rates [31]. This model generated theoretical results indicating that contractile VSMCs generate the vasomotion wave and spatiotemporal contractile oscillations of VSMCs. Further, the VSMC model showed more appropriate amplitude and wavelength parameters for brain fluid drainage at physiologically significant flow rates compared to the arterial pulse model [31]. These results suggest that the vasomotion stimulated by the contraction and relaxation cycle of VSMCs acts as the motive force of the IPAD pathway [31].

A recent experimental study addressed the role of VSMC-stimulated vasomotion using the fluorescent tracer dextran and in vivo two-photon microscopy in awake head-fixed mice [32]. The authors analyzed the role of vasomotion in solute clearance along vessels from the brain in wild-type and amyloid precursor protein (APP)/presenilin 1 (PS1) mice. APP/PS1 mice are a model of CAA/AD that expresses a mutant human APP gene harboring a Swedish mutation (K594N/M595L) and a mutant human PS1 (PS-1dE9) [32]. They demonstrated that the clearance rates of dextran extravasated into the brain were significantly associated with the vasomotion amplitude and that vasomotion was a major driving force for brain clearance [32]. Furthermore, CAA progression in the APP/PS1 mice resulted in a loss of VSMCs, which concomitantly reduced vascular reactivity and impaired solute clearance [32]. These findings highlight the notion that impaired VSMC activity results in cerebrovascular accumulation of Aβ and that VSMC contractile function could be an effective therapeutic target for CAA [31,32,33].

### 2.2. Effects of Cilostazol on the IPAD Pathway

The studies discussed so far suggest the functional significance of VSMCs in the IPAD pathway and that modulating VSMC function could be a useful approach to facilitate IPAD pathway activity. Cyclic adenosine monophosphate (cAMP) and cyclic guanosine monophosphate (cGMP) are second messengers in the VSMC intracellular signaling pathway that regulate various VSMC functions [34]. As negative regulators of cAMP and cGMP, cyclic nucleotide phosphodiesterases (PDEs) catalyze them rapidly. PDE-3 is a major cAMP-hydrolyzing PDE in VSMCs [34,35]. Selective PDE-3 inhibitors have been developed, including cilostazol. Cilostazol exhibits pleiotropic effects [36,37] such as the promotion of VSMC differentiation [38] and stabilization of the blood–brain barrier (BBB) function [39]. It has also been reported that cilostazol exerts protective effects against cellular stress [40].

To examine the functional role of cilostazol in suppressing cognitive impairment, a previous work from our group and colleagues analyzed PDE-3 expression levels in postmortem human brains. Immunohistochemical analyses revealed that the expression levels of PDE-3A, a PDE-3 isoform, were abnormally upregulated in cerebral VSMCs and showed a significant positive correlation with CAA severity [35]. To further these findings, we observed the effects of orally administered cilostazol on neuropathological alterations in a mouse model of CAA [35]. This model expresses a Swedish/Dutch/Iowa triple mutant of human APP that produces vasculotropic accumulation of Aβ_40_, in addition to Aβ_42_ [3,12,41]. Cilostazol facilitated Aβ drainage along the IPAD pathway, reduced Aβ accumulation in the brain, and prevented cognitive impairment [35]. Notably, cilostazol protected the cerebral VSMCs and pericytes from cellular injury/degeneration, which was quantified by vacuolization and intracellular organelle damage [35]. Accordingly, these findings suggest that cilostazol exhibits protective effects on the neurovasculature system and modulates VSMC function. This could promote IPAD pathway function and reduce Aβ deposition in the brain, thereby suppressing cognitive decline in CAA mice models [35,36,37].

### 2.3. Clinical Findings Obtained by Cilostazol Administration

Accumulating evidence has indicated that cilostazol shows clinically significant, beneficial effects in suppressing cognitive decline (Table 1) [42,43,44,45,46,47]. Intervention studies using a relatively small number of participants revealed that cilostazol exhibited protective effects against cognitive decline in AD patients [42,43]. Furthermore, retrospective studies have shown that cilostazol beneficially affected cognitive function in patients with mild cognitive impairment (MCI) [44] and those with mild dementia taking the anti-dementia drug donepezil [45]. Favorable effects of cilostazol for reducing the risk of cognitive decline in AD patients prescribed acetylcholinesterase inhibitors were also reported [46]. In addition, a 24-week, randomized, placebo-controlled study was recently conducted to examine the efficacy of cilostazol add-on therapy in AD patients with white matter lesions receiving donepezil [47]. The authors found that cilostazol maintained cerebral glucose metabolism. Further, a higher glucose metabolism was positively correlated with the improvement in cognitive function. These results suggest that cilostazol could preserve cerebral glucose metabolism integrity, which might contribute to improving or protecting cognitive function [47]. Randomized placebo-controlled studies with a larger sample size and longer study periods are required to further validate the effects of cilostazol [47]. One such study started in 2015 as a phase II, 96-week, randomized, placebo-controlled trial to determine whether cilostazol contributes to the preservation of cognitive function in patients with MCI and can prevent conversion to dementia: the Cilostazol for prevention of conversion from MCI to dementia (COMCID) study [48]. The results will be released in 2021 and will allow the field to gain significant understanding regarding the therapeutic potential of cilostazol [37].

## 3. Novel Therapeutic Potential of Taxifolin

### 3.1. Inhibitory Effects of Taxifolin on Aβ Aggregation

Natural bioactive molecules have been a topic of great interest because some may display preventive or therapeutic potential against cognitive decline. The brain has a high rate of oxidative metabolism relative to other tissues, making it particularly vulnerable to oxidative damage during aging [49]. Oxidative stress may cause cognitive impairment, so antioxidants could have beneficial effects when taken orally [49]. Considering this, extensive studies screened and identified a variety of antioxidants from plant extracts [49]. One of these is taxifolin, a flavonoid found in various plants such as milk thistle, onions, and Douglas fir bark [12,50]. This is an advantageous target to study because the biochemical and safety profiles of taxifolin have been established [12,51,52].

In addition to taxifolin’s antioxidative properties, multiple pharmacological activities have been reported [12,50]. Of importance for CAA/AD pathogenesis, taxifolin has inhibitory effects on Aβ aggregation in vitro. Using wild-type Aβ_42_ or mutant Aβ_42_ with substituted amino acids, systematic studies identified the mechanisms underlying taxifolin inhibition of Aβ_42_ aggregation. The *o*-quinone structure in the B-ring of taxifolin reacts with Lys16 and Lys28 of Aβ_42_. This generates taxifolin–Aβ_42_ adducts that inhibit Aβ_42_ aggregation because these Lys residues are located in the β-sheet region of Aβ_42_ [53,54]. Therefore, these reactions contribute to the suppression of toxic Aβ_42_ aggregates [53,54].

We further examined whether taxifolin exerts inhibitory effects on Aβ_40_ aggregation in vitro using the thioflavin T fluorescence assay [3]. The results revealed that taxifolin significantly inhibited Aβ_40_ aggregation [3]. Transmission electron microscopy images confirmed that taxifolin suppressed Aβ_40_ fibril formation [3]. Although additional studies are needed to elucidate the molecular mechanism of action of taxifolin in inhibiting Aβ_40_ aggregation, our results demonstrated novel inhibitory effects of taxifolin on Aβ_40_ fibril formation [3].

### 3.2. Effects of Taxifolin in a Mouse Model of CAA

Taxifolin exerts antioxidant properties and suppresses Aβ fibril formation in vitro. However, only a small amount of taxifolin passes the BBB when taken orally [3,50]. We therefore investigated the in vivo effects of orally administered taxifolin using an aforementioned mouse model of CAA [3,12,35,41] fed standard chow or chow containing taxifolin [3,4]. The filter trap assay and enzyme-linked immunosorbent assay (ELISA) revealed that Aβ aggregation levels in the brain were significantly decreased in the taxifolin group compared to control, consistent with the in vitro experiments [3]. Furthermore, laser speckle flowmetry demonstrated that taxifolin improved cerebral blood flow. This may have been related to the immunohistochemical and ELISA results indicating that Aβ levels were decreased in the brain and increased in the blood [3]. We further showed that taxifolin prevented cognitive decline in a spatial reference memory test [3]. Taxifolin inhibited Aβ aggregation and promoted Aβ drainage from the brain, thereby inhibiting cognitive decline in a CAA mouse model [3]. These results indicate that taxifolin might facilitate Aβ drainage along the aforementioned IPAD pathway and reinforce the significance of Aβ drainage from the brain in neuroprotection [3]. Future studies should elucidate the mechanism of action of taxifolin in the IPAD pathway by analyzing its molecular action on VSMCs and vascular endothelial cells.

We further performed biochemical and molecular biological analyses to investigate the intracerebral effects of taxifolin in a CAA mouse model. Despite taxifolin’s limited BBB permeability, orally administered taxifolin significantly reduced the levels of cerebral lipid peroxidation, an oxidative tissue damage marker, in the brain. Further, cerebral expression levels of oxidative-stress-responsive genes were decreased in the taxifolin group [4]. Taxifolin also inhibited the production of Aβ in the brain, potentially through the suppression of the ApoE–ERK1/2–APP axis [4], which mediates Aβ transcription and secretion in the brain [55,56]. Additionally, immunohistochemical analysis revealed that taxifolin reduced activated microglia accumulation in the brain [4]. Interestingly, the expression levels of TREM2, a cell surface receptor exclusively expressed on microglia in the brain and implicated in neurodegenerative diseases [18,19], were positively correlated with the exacerbation of brain inflammation. Taxifolin improved brain inflammation, reduced TREM2 expression levels, and reduced the accumulation of TREM2-expressing cells in the brain [4]. In line with these neuroprotective effects, taxifolin reduced the indicators of apoptotic cell death in the brain [4]. Therefore, despite its limited BBB permeability, orally administered taxifolin exhibited beneficial in vivo pleiotropic effects that can help in preventing cognitive impairment in a CAA mouse model [4]. Additional studies are required to elucidate the mechanisms underlying taxifolin’s beneficial effects irrespective of its limited BBB permeability. These findings demonstrate the novel therapeutic and preventive potentials of taxifolin.

### 3.3. Effects of Taxifolin in a Mouse Model of AD

Similar to CAA, taxifolin reportedly exerted in vivo beneficial effects on AD pathogenesis in a mouse model of AD [57]. This AD model is based on bilateral Aβ_42_ injection into hippocampal regions [57]. The authors treated mice with taxifolin (intraperitoneal injections) and examined its effects on neuroinflammation and cognitive dysfunction [57]. Quantitative analysis by ELISA revealed that taxifolin significantly decreased the levels of prostaglandin E2, a proinflammatory mediator, in the hippocampus. There was also a significant reduction in the level of cytosolic phospholipase A_2_, one enzyme for prostaglandin E2 production [57]. Furthermore, behavioral tests assessing recognition and spatial memory in these mice demonstrated protective effects of taxifolin against cognitive impairment [57]. In vitro experiments further showed that taxifolin prevented Aβ_42_-induced cell death in a human neuroblastoma cell line [57]. Additionally, taxifolin suppressed the loss of dendritic filopodium and synapses caused by Aβ_42_ incubation in primary cultures of mouse hippocampal neurons [57]. These results suggest that taxifolin can effectively suppress the development and progression of cognitive impairment in a mouse model of AD [57].

Taken together, this basic and preclinical evidence highlights the beneficial effects of taxifolin. The safety of using taxifolin has already been established [51,52], so clinical trials to examine the efficacy of taxifolin for treating CAA and AD are of significance.

## 4. Strategies for Inhibiting Aβ Production

### 4.1. Clinical Trials Targeting β-Site APP Cleaving Enzyme-1

Thus far, many studies have tried to develop therapeutic strategies for AD by inhibiting Aβ production by targeting Aβ-generating enzymatic processes. One method is to inhibit the β-site APP cleaving enzyme-1 (BACE1, also called β-secretase), which is involved in initiating Aβ generation [58,59]. Clinical trials targeting BACE1 have largely failed possibly because of inadequate classification of AD stages and adverse events [60]. The adverse outcomes may have been associated with inhibitory effects on normal synaptic function [61] and off-target effects on protein processing [62].

A novel BACE1 inhibitor, CNP520, was recently developed. Its efficacy was examined in the clinical trial titled the Alzheimer’s Prevention Initiative Generation Program, which enrolls presymptomatic individuals at risk for AD [58,60,63]. This novel inhibitor has demonstrated preclinical and preliminary clinical safety profiles and favorable effects, such as reductions in brain Aβ levels [58]. However, a recent data assessment in this clinical trial found worsening in some measures of cognitive function, leading to the discontinuation of the clinical program [64]. The detailed results and associated mechanisms regarding the observed adverse events warrant further investigation.

### 4.2. BACE1 Inhibition and CAA

Not much is known about the efficacy of BACE1 inhibition in CAA compared to AD. A recent study addressed this issue using a mouse model of CAA, APPDutch mice overexpressing a mutated human APP gene [6]. Electrochemiluminescence-linked immunoassay results revealed that the oral administration of the BACE1 inhibitor NB-360 reduced Aβ_40_ and Aβ_42_ levels in CSF and the brain. This suggests that NB-360 has in vivo suppressive effects on Aβ production [6]. Histological analyses further showed that NB-360 prevented clustering of activated microglia around vessels and suppressed the loss of vessel-associated SMCs [6]. In line with these beneficial effects on CAA-related pathologies, the frequency and severity of CAA were decreased by NB-360 administration [6]. These results suggest that BACE1 inhibition effectively prevents CAA, although the effects of NB-360 on cognitive function were not addressed [6]. These findings also indicate that the inhibition of Aβ production could suppress the development and progression of CAA. This reinforces the theory that Aβ is a cause, rather than a by-product, of cognitive dysfunction in CAA- and AD-associated pathology. The disappointed results of clinical trials targeting BACE1 for AD indicate the need for careful consideration of the therapeutic potential of BACE1 inhibition for CAA. However, as noted by the authors, Aβ-reducing trials might be effective in individuals at risk for CAA [6].

### 4.3. Supperessive Effects of Cilostazol and Taxifolin on BACE1 Expression

Several signaling molecules and their mechanisms of action have been previously reported for modulating BACE1 expression. In neurons, constitutive activation of the Janus kinase 2 (JAK2)/signal transducer and activator of transcription 1 (STAT1)-related signaling pathway mediates BACE1 expression [65]. Activation of STAT3 [66] and the stress-associated transcription factor nuclear factor-kappa B (NF-κB) [67] also stimulate BACE1 expression. Accordingly, these pathways could be potential targets to reduce Aβ production by attenuating BACE1 expression. A previous study used biochemical and immunocytochemical analyses to investigate whether the aforementioned bioactive molecules cilostazol and/or taxifolin affected these signaling components and influenced BACE1 expression in the mouse neuroblastoma cell line, N2a, which stably expresses the human APP Swedish mutant gene [68]. They found that cilostazol and taxifolin increased the expression levels and activity of SIRT1 [68], a deacetylase suggested to have protective effects against neurodegenerative diseases [69]. In turn, this suppressed JAK2-mediated STAT3 activation, which further resulted in NF-κB signaling inhibition, thereby leading to suppressed BACE1 expression [68]. They also demonstrated that the suppressive effects of cilostazol and taxifolin on JAK2, STAT3, and NF-κB activation were synergistic when these treatments were combined [68]. Therefore, it is suggested that cilostazol and taxifolin target common and non-common molecules across multiple pathways that mediate BACE1 expression. This results in the synergistic suppressive effects on BACE1 expression. As described previously, both agents have established safety profiles, and preclinical studies have shown the beneficial effects of the monotreatment with these agents in a mouse model of CAA [3,4,35]. Clinical studies have also reported favorable effects of cilostazol [42,43,44,45,46,47]. Thus, concurrent treatment with cilostazol and taxifolin may be a more efficacious potential therapeutic agent in clinical studies [68].

## 5. Potential Neuroprotective Effects of Antioxidants

### 5.1. Preclinical Findings Obtained by Twendee X Administration

It has been reported that chronic cerebral hypoperfusion (CCH) induces oxidative stress and neuroinflammation, potentially contributing to the progression of neurodegenerative diseases [70,71]. Thus, recent studies have examined whether an antioxidant supplement, Twendee X (TwX), exhibits beneficial effects in a mouse model of AD with CCH [72,73]. TwX contains the antioxidants coenzyme Q10, niacin amid, L-cystine, ascorbic acid, succinic acid, fumaric acid, L-glutamine, and riboflavin [72,73,74]. One study administered TwX by oral gavage to APP23 mice, which overexpress the human APP Swedish mutant gene, that were treated with ameroid constrictors on bilateral common carotid arteries to induce CCH development [72,73]. Immunohistochemical analyses revealed that TwX reduced oxidative stress levels in the brain [72]. Furthermore, it reduced parenchymal and vascular Aβ deposition levels, microglial activation, and neuroinflammation in the brain [72]. In line with these neuroprotective effects, TwX suppressed neuronal loss and prevented cognitive dysfunction [72]. Although the molecular mechanisms underlying TwX’s effects remain unclear, the authors discussed that oxidative stress stimulates the proinflammatory pathway [75] and the signaling pathway for APP processing [76], which includes increased BACE1 expression and concomitant Aβ generation [77]. Therefore, the potent antioxidant TwX could scavenge ROS and suppress the activation of these pathways [72]. They further performed immunohistochemical analyses on the brains of these mice and found that TwX reduced phosphorylated Tau levels, phosphorylated α-synuclein accumulation, and expression of matrix metalloproteinase-9, which is related to BBB destruction [73]. These results suggest that TwX has pleiotropic neuroprotective effects in a mouse model of AD with CCH [72,73]. However, the mechanistic details underlying TwX’s beneficial effects warrant further investigation.

### 5.2. Effects of TwX on Patients with MCI

On the basis of the described preclinical findings, the authors conducted a randomized, double-blind, placebo-controlled clinical trial to examine the efficacy of TwX in patients with MCI [74]. They assessed cognitive function using the Mini-Mental State Examination and Hasegawa Dementia Scale-revised scores at baseline and six months and compared the placebo (n = 37) and TwX (n = 41) groups [74]. Results revealed that cognitive function was improved in patients in the TwX group compared to the placebo group following six months of intervention [74]. TwX antioxidant therapy might contribute to the prevention of or delay of the progression of MCI to AD dementia [74]. Notably, the mechanisms of TwX’s clinical benefits have not been elucidated, in part, because the central and peripheral effects of TwX on mechanism-related indices have not been examined in these patients. Potential mechanisms include the levels of oxidative stress, Aβ, and inflammation. It also remains unclear whether TwX beneficially influences the IPAD pathway and promotes Aβ drainage from the brain. These issues must be addressed using a larger sample size and a longer study period to corroborate these findings and provide a rationale for TwX’s clinical benefits. This may contribute to the development of safe and effective treatments for MCI, CAA, and AD.

## 6. Glycation and Aβ Dynamics

### 6.1. Cytotoxicity of Glycated Aβ and Efficacy of Anti-Glycation Agents

Advanced glycation end products (AGEs) are stable end products of a non-enzymatic post-translational glycation reaction [78,79]. In vivo, AGEs are mainly formed through the reaction of proteins with dicarbonyl compounds, such as methylglyoxal (MGO), a by-product of glycolysis [78]. When plaque-enriched fractions of AD brains were analyzed, higher AGE levels were detected compared to those in age-matched controls [80]. Thus, Aβ seems to be highly glycated in AD brains [80]. To clarify the characteristics of glycated Aβ, another study prepared glycated Aβ_42_ in vitro by incubating Aβ_42_ with MGO and examined the effects of the reaction products on mouse primary hippocampal neurons [81]. Biochemical and cell biological analyses revealed that glycated Aβ_42_ increased apoptosis and reduced cell viability compared to non-glycated Aβ_42_ [81]. In addition, the authors subcutaneously administered aminoguanidine (AG), an AGE formation inhibitor, to Tg2576 mice, which overexpress a human APP mutant gene [81]. Biochemical analysis showed that AG reduced glycated Aβ_42_ levels in the brain compared to control [81]. Furthermore, synaptic and memory-associated protein levels were decreased in the brain of the control group, but they were restored in the AG group [81]. In line with these neuroprotective effects, mice in the AG group showed improved cognitive function compared to the control group [81]. These results suggest an increased detrimental profile of glycated Aβ_42_ compared to non-glycated Aβ_42_ in AD pathogenesis. Therefore, glycated Aβ_42_ might be an effective therapeutic target for AD [81].

### 6.2. Potential Mechanisms Underlying Increased Cytotoxicity of Glycated Aβ

As described above, glycated Aβ demonstrates greater toxicity than non-glycated Aβ [81]. To address the associated mechanisms, a recent study reacted Aβ_40_ or Aβ_42_ with MGO in vitro and then analyzed the formation of aggregates and fibers and its kinetics using the thioflavin T assay and atomic force microscopy [79]. The authors found that MGO-induced glycation reduced the aggregation speed of Aβ_40_ and Aβ_42_. Consequently, formation of mature fibers was slowed down. This would suggest the accumulation of Aβ oligomers because the slower fiber formation process could reflect a stabilizing effect on the oligomeric state [79]. Importantly, intermediate aggregates of Aβ, such as oligomers, are particularly cytotoxic and closely implicated in AD pathogenesis [7,8,79,82]. Thus, Aβ glycation could result in the extended presence of the toxic oligomeric form, thereby leading to higher toxicity compared to non-glycated Aβ [79].

It has been reported that MGO can react with Arg and Lys residues in proteins [78,79,83]. To further clarify the pathological significance of glycated Aβ_42_, a recent study examined the characteristics of three glycated Aβ_42_ variants compared to non-glycated Aβ_42_. The glycated variants possessed site-specific glycation at Lys16, Lys28, or Lys16 and Lys28 [84]. Transmission microscopy and in silico modeling revealed that double Aβ_42_ glycation at Lys16 and Lys28 reduced free energy change and destabilized fibril structures, thereby increasing the rate of Aβ_42_ aggregation [84]. They also showed that a single glycation at Lys16 slowed down fibril formation, whereas a single glycation at Lys28 had no significant effect [84]. These results suggest that double-glycated (Lys16 and Lys28) and single-glycated (Lys16) Aβ_42_ tended to maintain a low rate of fibrils and a high rate of oligomers, whereas single-glycated (Lys28) and non-glycated Aβ_42_ had a high rate of fibrils and a low rate of oligomers [84]. Non-glycated and single-glycated Aβ_42_ exhibited cytotoxic effects on the retinoic-acid-differentiated human neuroblastoma cell line SH-SY5Y, which was assessed using the 3-(4,5-dimethylthiazol-2-yl)-2,5-diphenyltetrazolium bromide assay [84]. Notably, double-glycated Aβ_42_ did not show significant cytotoxic effects, even though it had a depolarized mitochondrial membrane potential [84]. The authors speculated that this depolarization by double-glycated Aβ_42_ would activate the pathway that mediates selective degradation of dysfunctional mitochondria, which concomitantly maintains a healthy pool of mitochondria and neuronal function [84]. Furthermore, findings from other studies [79,81] have indicated that it is possible for a mixture of these glycated Aβ variants to produce synergistic detrimental effects on neuronal functions.

As described previously, Lys16 and Lys28 residues are also targeted by taxifolin [53,54]. Thus, taxifolin could exhibit the suppressive effects on the cytotoxic oligomerization of Aβ in glycated and non-glycated conditions.

Epidemiological studies have reported that diabetes is a high-risk factor for AD and vascular cognitive impairment [85,86,87]. One reason for this could be the increased formation of glycated Aβ [79,81]. Unlike glycated Aβ_42_, the pathological significance of glycated Aβ_40_ in CAA and AD has not been elucidated. However, since the glycation of Aβ_40_ should increase the toxic oligomeric state similar to Aβ_42_ [79], glycated Aβ_40_ may have more toxic effects than non-glycated Aβ_40_. Inhibiting Aβ_40_ and Aβ_42_ glycation could therefore reduce the toxic intermediate oligomeric form. In addition, Aβ glycation leads to the formation of the AGE-cross-linked amyloid oligomer, which cannot be converted to a less toxic amyloid fibril [88]. Therefore, promoting mature fibril formation by inhibiting Aβ glycation could also decrease the levels of toxic intermediate aggregates [88]. In this respect, approaches to eliminate toxic intermediate structures by targeting Aβ glycation reactions may have greater therapeutic potential than previously thought in diseases characterized by Aβ accumulation.

## 7. A Novel Molecular Target for BBB and Cerebrovascular Integrity

BBB breakdown and cerebrovascular dysfunction have been strongly implicated in AD pathogenesis [89,90]. Accordingly, mechanisms for maintaining the BBB and cerebrovascular integrity could be effective therapeutic targets for CAA and AD. A recent study meticulously demonstrated the novel protective effects of a disintegrin and metalloprotease with a thrombospondin type I motif, member 13 (ADAMTS13) against CAA and AD [91]. ADAMTS13 negatively regulates the multimeric size and activity of von Willebrand factor (VWF), an adhesive circulating ligand that is produced in activated and injured endothelial cells and promotes vascular leakage [92].

In light of the vascular protective effects of ADAMTS13, the authors investigated its potential role in preventing CAA and AD by deleting the ADAMTS13 gene in APPPS1 mice, which express a chimeric mouse/human APP (Mo/Hu APP 695swe) and a mutant human presenilin 1 (PS1-dE9) [91]. On the basis of the biochemical analyses and in vivo multiphoton image assays, they found that ADAMTS13 deficiency in APPPS1 mice reduced the expression levels of proteins for tight or adherens junctions. This resulted in early and progressive BBB damage that induced BBB leakage [91]. ADAMTS13 deficiency also induced microvascular and cerebral blood flow reductions in APPPS1 mice [91]. Furthermore, ADAMTS13 deficiency increased Aβ_40_ and Aβ_42_ levels in the brain and elevated CAA and parenchymal plaque levels [91]. In line with these pathological changes, ADAMTS13 deficiency cognitive function was more exacerbated in compared to control APPPS1 mice [91]. Importantly, although ADAMTS13 deficiency had no significant effect on Aβ production or processing in the brain, it reduced the expression levels of Aβ efflux transporters in the brain and Aβ_40_ and Aβ_42_ levels in the blood [91]. These results suggest that ADAMTS13 deficiency reduced BBB-mediated Aβ clearance in the brain, thereby causing cognitive impairment [91].

Notably, virus-mediated injection of ADAMTS13 into the hippocampi of APPPS1 mice with vascular damage, plaque deposition, and cognitive deficits beneficially reversed a diverse array of ADAMTS13 deficiency-related phenotypes, including cognitive impairment [91]. Therefore, ADAMTS13 stimulation or VWF regulation could have pleiotropic benefits in CAA and AD. However, the detailed pathological roles of ADAMTS13 and VWF in patients with CAA and AD have not been elucidated [91]. Furthermore, these findings highlight the significance of Aβ drainage through the cerebrovasculature system for the prevention and treatment of CAA and AD. In this context, it is important to address whether a functional relationship exists between the ADAMTS13–VWF axis and cilostazol and taxifolin treatment.

## 8. Soluble TREM2 as a Potential Predictive Marker for Dementia Incidence

It has been reported that pathological changes in the brain, such as increased Aβ levels, may precede dementia diagnosis by several years [93]. However, there are currently no established markers to predict the incidence of dementia. A population-based longitudinal study recently addressed this issue by focusing on the potential role of TREM2 [17]. TREM2 is a microglia-specific cell surface molecule in the brain that releases its soluble form (soluble TREM2, sTREM2) into the extracellular space [18,19]. Although sTREM2 levels in CSF have been implicated in neuroinflammation and neurodegeneration [19], the pathophysiological significance of blood sTREM2 in cognitive impairment remains unclear. To clarify the clinical significance of serum sTREM2 levels, we and our colleagues examined its relationship with dementia risk by prospectively following 1349 elderly Japanese community residents without dementia for 10 years [17]. The results demonstrated that higher levels of serum sTREM2 were significantly associated with a higher risk of developing all-cause dementia, AD, and vascular cognitive impairment [17]. Furthermore, the inclusion of serum sTREM2 levels in a model of known potential risk factors for dementia significantly improved the ability to predict the development of dementia [17]. Therefore, serum sTREM2 levels may be a novel marker for dementia incidence in the general elderly population [17]. A recent cross-sectional study also showed that elevated serum sTREM2 levels were significantly associated with an increased risk of cognitive impairment in non-obese patients with type 2 diabetes [94]. Accordingly, these findings suggest that serum sTREM2 is a potential blood-based biomarker to predict the incidence of dementia [17,94].

To date, it remains unclear whether serum sTREM2 levels are associated with a future risk of CAA. However, CAA is closely implicated in the pathogenesis of AD and vascular dysfunction [5]. It has also been suggested that a pathological relationship exists between serum sTREM2 levels and central/peripheral inflammation, resulting in vascular damage [17,94]. In this respect, serum sTREM2 might also be predictive of CAA development. Further studies are required to elucidate the mechanisms underlying the relationship between serum sTREM2 levels and future incidence of dementia.

## 9. Future Perspectives

**Figure 1:**
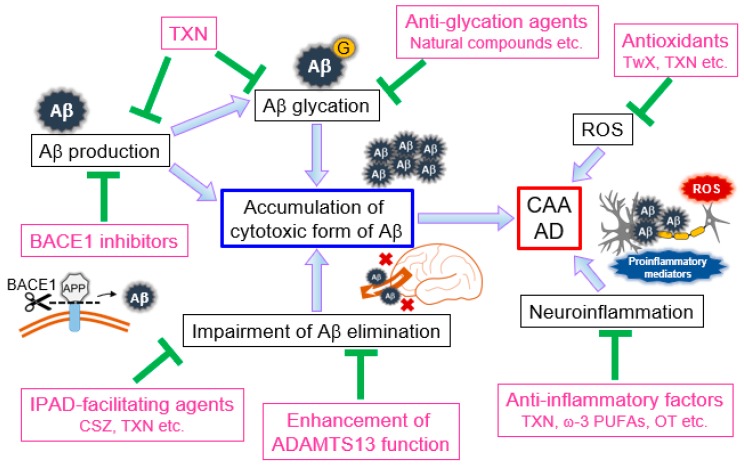
Pathological implications of amyloid-β (Aβ), reactive oxygen species (ROS), and neuroinflammation and their role in potential therapeutic approaches for cerebral amyloid angiopathy (CAA) and Alzheimer’s disease (AD). Aβ produced in the brain aggregates to form fibrils, with intermediate oligomers being particularly cytotoxic. When Aβ is glycated, the products result in the persistence of cytotoxic oligomers. Dysfunction of the Aβ elimination system also results in Aβ accumulation in the brain. This Aβ-related pathology can result in the injury of cerebrovascular endothelial cells, VSMCs, and neurons, thereby promoting development and progression of CAA and AD. ROS and neuroinflammation are also cytotoxic factors closely implicated in CAA and AD pathogenesis. BACE1 inhibitors that inhibit Aβ production have been developed. Anti-glycation agents could reduce the accumulation of cytotoxic Aβ oligomers. Agents that facilitate the formation of mature fibers would also be effective. Taxifolin (TXN) has pleiotropic beneficial effects, including the suppression of the production and glycation of Aβ. The Aβ elimination system is also an effective target to reduce detrimental Aβ accumulation. Agents that facilitate the IPAD pathway or enhance ADAMTS13 function, antioxidants, and anti-inflammatory mediators may also exhibit protective effects against CAA and AD. Aβ, amyloid-β; AD, Alzheimer’s disease; ADAMTS13, a disintegrin and metalloprotease with thrombospondin type I motif, member 13; BACE1, β-site amyloid precursor protein cleaving enzyme-1; CAA, cerebral amyloid angiopathy; CSZ, cilostazol; IPAD, intramural peri-arterial drainage; OT, oxytocin; PUFAs, polyunsaturated fatty acids; ROS, reactive oxygen species; TwX, Twendee X; TXN, taxifolin.

Recent remarkable advances in CAA and AD research have accelerated our understanding of pathogenesis, therapeutic approaches, and predictive markers. This work has provided significant evidence regarding novel therapeutic strategies for these diseases (Figure 1). Aβ is considered one of the major causes of dementia-related diseases, and accumulating evidence has revealed an emerging significant role of the Aβ drainage system in suppressing the development and progression of CAA and AD. Unfortunately, currently available treatment options do not necessarily reverse disease progression [59]. Excitingly, a recent preclinical study showed that promoting Aβ drainage from the brain by restoring BBB and cerebrovasculature integrity improved CAA- and AD-related pathologies, including cognitive decline, even after the disease had developed [91]. Although the mechanism of these effects remains unclear, it is possible that restoring the Aβ drainage system can result in remodeling or regeneration of lost neural circuits. This would further support the theory that improving IPAD pathway integrity can reverse the disease process. In this respect, cilostazol and taxifolin are potential therapeutics with pleiotropic benefits, including potential IPAD pathway facilitation. Therefore, future clinical studies to assess the efficacy of these agents would reveal novel insights regarding the development of novel therapeutic strategies for CAA and AD.

Despite continuous efforts to offer antioxidants as potential therapeutic agents for AD, the evidence regarding their ability to slow down AD progression has been inconclusive [95]. Therefore, it is notable that the antioxidant supplement TwX showed beneficial effects in recent preclinical and clinical studies [72,73,74], which supports antioxidative treatment for AD. However, the effectiveness of antioxidants, particularly for cognitive decline, seems to be highly sensitive to various factors. One possible reason for this is that reliable biomarkers for oxidative stress have not been established, so it is difficult to distinguish between non-responders and responders by measuring oxidative stress within individuals [95]. Another possibility is the differing bioavailability of antioxidants between individuals. For example, vitamin E bioavailability is influenced by inherited and environmental factors, including age, gender, dietary pattern, and genetic polymorphisms [95].

Another reason for the concern regarding antioxidant treatment is the potential impact of antioxidants on cancer patients receiving ROS-dependent chemotherapy or radiotherapy [96]. Although some studies have reported no significant effect of antioxidants on cancer treatments, others have reported that antioxidants’ ROS-scavenging properties can protect tumor cells from oxidative damage induced by chemotherapy or radiotherapy, thereby negatively affecting the outcome of these therapies [96]. Thus, antioxidants would need to be administered with careful consideration regarding individual health conditions.

A critical aspect for neuroprotection is to reduce the levels of Aβ oligomers, which are particularly cytotoxic intermediates in Aβ dynamics [7,8,79,81,82,84]. Aβ glycation causes Aβ oligomer accumulation. Therefore, the inhibition of Aβ glycation could be an effective therapeutic approach for CAA and AD. Although effective therapeutic agents that inhibit Aβ glycation have not been developed, a number of natural compounds, including taxifolin, exert inhibitory effects on the glycation reaction [97,98]. Therefore, future studies may identify a molecule in these natural compounds that can be used or modified for CAA and AD treatment. Furthermore, agents that promote processing of intermediate oligomers into mature fibrils could reduce the number of cytotoxic oligomers.

Notably, a recent study demonstrated that cell-surface-mediated Aβ self-assembly is a novel mechanism underlying Aβ aggregation [99]. When an Aβ monomer interacts with the cellular membrane, conformation of the Aβ monomer is changed to an aggregation-prone form [99]. A dimer is formed when a second monomer undergoes a conformational change on the cell surface [99]. Continued on-surface monomer docking results in the formation of oligomers, which are released from the surface, ultimately stimulating disease-related pathways [99]. Thus, this newly identified mode of action may also be targeted for novel therapeutics, including factors involved in membrane composition and membrane–Aβ interaction [99].

Another significant target for intervention is the inflammation in the brain [13]. Microglia are the main cell type that mediates the pathological relationship between neuroinflammation and neurodegeneration [13]. Therefore, regulation of microglial activity could be critical in suppressing neuroinflammation. TREM2 is highly regarded as a potential key molecule in neurodegenerative disease pathogenesis. Whether TREM2-expressing microglia are beneficial or detrimental remains inconclusive, but recent findings suggest differential roles of these cells across different stages of disease progression [19]. Stimulation of TREM2 might be favorable in the early stage, whereas blockage of TREM2 might be promising at the later stage to alter the disease course [19]. Although further studies are required to define the role of TREM2-expressing microglia in CAA and AD, accumulating evidence regarding TREM2 and sTREM2 indicates its potential as an effective therapeutic target for these diseases.

A recent study reported the novel functional significance of purinergic receptor P2RY12-expressing microglia that may elucidate the relationship between neuroinflammation and microglia [100]. P2RY12-expressing microglia initiate chemotaxis to the site of damaged and dying cells in response to adenosine triphosphate and diphosphate released from the injured cells [100]. Upon reaching the targets, they reduce P2RY12 expression levels by shifting the microglial phenotype to a proinflammatory state [100]. Proteomic analysis comparing areas where P2RY12-positive and P2RY12-negative microglia are distributed could identify novel factors involved in neuroinflammation [100].

Various factors have been reported to exert beneficial anti-inflammatory effects. One of these factors is minocycline, which is a tetracycline derivative with anti-inflammatory properties that rapidly crosses the BBB [101,102,103]. Minocycline exerts inhibitory effects on microglial activation, thereby leading to neuroprotection and suppression of cognitive impairment in a mouse model of CAA [101,104] and AD [102,103,105]. In humans, unfortunately, a recent randomized clinical trial reported that 24 months of minocycline treatment did not delay the progression of cognitive or functional impairment in patients with mild AD [106]. The authors speculated that the absence of clinical benefits in this trial could be ascribed to (i) the potentially lesser pathological significance of neuroinflammation in mild AD, (ii) potentially insufficient doses of minocycline to afford efficacy (of note, this agent would not result in apparent benefits even if higher doses were used, because of its tolerability), and (iii) potential clinical benefits that would be too small for detection [106]. In addition to minocycline, there is an emerging list of factors with therapeutic potential, including taxifolin [4,57], ω-3 polyunsaturated fatty acids [107,108], oxytocin [109,110], and exercise [111,112]; therefore, concurrent treatment might be beneficial, as suggested recently [113]. Further investigation to elucidate the clinical significance and effectiveness of these factors may provide effective treatment options for CAA and AD.

To date, cytotoxic mediators per se have been a topic of great interest, in terms of the notion of prevention of neuronal injury, for neuroprotection. Conversely, recent advances in research on CAA and AD have shown that rather than a single dominant factor, such as Aβ, multifactorial pathways are implicated in the pathogenesis of these diseases [114]. In this respect, the finding that vascular lesions are the earliest pathological changes that precede Aβ accumulation was notable, thereby suggesting vascular integrity as a key target for the prevention and/or treatment of CAA and AD [36,114]. Furthermore, vascular lesions are related to aging and metabolic diseases, including type 2 diabetes and hypertension, and arteriosclerosis causes the impairment of Aβ elimination from the brain [115]. Thus, anti-diabetic drugs and cardiovascular drugs might be effective as potential therapeutic agents for CAA and AD [114]. In light of this possibility, drug repositioning studies for AD using anti-diabetic drugs and cardiovascular drugs, including cilostazol, that target vascular functions are currently ongoing [114]. Bioactive molecules, such as taxifolin, that enhance the IPAD pathway will also be effective. Overall, the neurovascular approach is now highly regarded as the most promising therapeutic strategy for CAA and AD [36].

In conclusion, recent studies on CAA and AD have provided deeper understanding of the pathogenesis of these multifactorial and complicated diseases. This work has highlighted the significance of eliminating Aβ from the brain and modulating other factors, including Aβ production, ROS, Aβ aggregation, and neuroinflammation. Many potential therapeutic agents demonstrating pleiotropic beneficial effects that mediate disease-associated pathways have been identified and explored. Future basic and clinical research targeting these pathways will contribute to the development of novel therapeutic strategies for CAA and AD.

## Figures and Tables

**Table 1:** Effects of cilostazol on cognitive function in patients with AD or mild, moderate, and/or severe dementia.

Authors (Publication Year)	Study Design	Subjects Treated with Cilostazol	Period	Measurement	Results ^1^
Arai et al. (2009 [42])	An intervention study	10 patients with moderate AD who had received donepezil	Mean follow-up: 7.6 months	MMSE	Improved cognitive function for 5–6 months
Sakurai et al. (2013 [43])	An intervention study	11 patients with possible AD and confirmed cerebrovascular disease lesions	6 months	MMSE; ADAS-Jcog; WMS-R logical memory-I; TMT-A	Maintained cognitive function, except for MMSE scores
Taguchi et al. (2013 [44])	A retrospective study	All cases treated with cilostazol and previously evaluated by MMSE (70 patients)	More than 6 months (Mean follow-up: control, 820 days; treated, 650 days)	MMSE	Improved cognitive function in patients with MCI, but not in those with normal cognitive function or dementia
Ihara et al. (2014 [45])	A retrospective study	69 patients with mild (n = 34) and moderate/severe (n = 35) dementia who had received donepezil	More than 1 year (Mean follow-up: control, 30.4 months; treated, 28.6 months)	MMSE	Maintained cognitive function in patients with mild dementia, but not in those with moderate/severe dementia
Tai et al. (2017 [46])	A retrospective study	30 patients with AD who had received AChEIs	1 year	MMSE; CDR-SB	Reduced risk of deterioration of cognitive function
Lee et al. (2019 [47])	An intervention study	18 AD patients with white matter lesions who had received donepezil	24 weeks	MMSE; ADAS; ADCS-ADL; CDR-SB	Did not maintain cognitive function, but preserved regional cerebral glucose metabolism

^1^ Effects of cilostazol compared to treatment without cilostazol or at baseline. Abbreviations: AChEIs, acetylcholinesterase inhibitors; AD, Alzheimer’s Disease; ADAS, Alzheimer’s Disease Assessment Scale—cognitive subscale; ADAS-Jcog, ADAS Japanese version; ADCS-ADL, Alzheimer’s Disease Cooperative Study—Activities of Daily Living; CDR-SB, clinical dementia rating sum of boxes; MMSE, Mini-Mental State Examination; TMT-A, Trail Making Test A; WMS-R, Wechsler Memory Scale-Revised.

## Abbreviations

AD	Alzheimer’s disease
ADAMTS13	A disintegrin and metalloprotease with thrombospondin type I motif, member 13
Aβ	Amyloid-β
APP	Amyloid precursor protein
BACE1	β-site APP cleaving enzyme-1
BBB	Blood–brain barrier
CAA	Cerebral amyloid angiopathy
CSF	Cerebrospinal fluid
IPAD	Intramural peri-arterial drainage
MCI	Mild cognitive impairment
ROS	Reactive oxygen species
TREM2	Triggering receptor expressed on myeloid cells 2
TwX	Twendee X
VSMCs	Vascular smooth muscle cells
